# Influence of extreme weather and meteorological anomalies on outbreaks of influenza A (H1N1)

**DOI:** 10.1007/s11434-012-5571-7

**Published:** 2012-12-29

**Authors:** Hong Xiao, HuaiYu Tian, XiaoLing Lin, LiDong Gao, XiangYu Dai, XiXing Zhang, BiYun Chen, Jian Zhao, JingZhe Xu

**Affiliations:** 1 College of Resources and Environmental Science, Hunan Normal University, Changsha, 410081 China; 2 Hunan Provincial Center for Disease Control and Prevention, Changsha, 410002 China; 3 Changsha Municipal Center for Disease Control and Prevention, Changsha, 410001 China; 4 Peking University Health Science Center, Beijing, 100191 China

**Keywords:** influenza A (H1N1), meteorological anomaly, geographic information system, absolute humidity, SIR model

## Abstract

Biological experiments and epidemiological evidence indicate that variations in environment have important effect on the occurrence and transmission of epidemic influenza. It is therefore important to understand the characteristic patterns of transmission for prevention of disease and reduction of disease burden. Based on case records, we analyzed the environmental characteristics including climate variables in Changsha, and then constructed a meteorological anomaly susceptive-infective-removal (SIR) model on the basis of the results of influenza A (H1N1) transmission. The results showed that the outbreak of influenza A (H1N1) in Changsha showed significant correlation with meteorological conditions; the spread of influenza was sensitive to meteorological anomalies, and that the outbreak of influenza A (H1N1) in Changsha was influenced by a combination of absolute humidity anomalous weather conditions, contact rates of the influenza patients and changes in population movements. These findings will provide helpful information regarding prevention strategies under different conditions, a fresh understanding of the emergence and re-emergence of influenza outbreaks, and a new perspective on the transmission dynamics of influenza.

In 2009, the global influenza A (H1N1) epidemics introduced a great burden of disease to China as well as to the rest of the world. In our opinion such disasters caused by the influenza pandemics may very well recur again in the future [1]. Since the beginning of the 20th century, there have been three influenza pandemics around the world together affecting millions of people [2,3]; the ‘Spanish flu pandemic’ (H1N1) of 1918 [4–7], the influenza A subtype H2N2 pandemic in 1957 [8], and the global pandemic caused by the influenza A subtype H3N2 in 1968 [9]. The outbreak and spread of influenza is a complex process, clearly influenced by the virulence of the influenza virus, the immunity of the host, and frequency of contacts between hosts. Research conducted in the last few decades has described a number of factors that influence the transmission and spread of influenza, including (1) mutation of the viruses and the fluctuating of population immunity levels, such as the emergence of new virus subtypes [10,11], changes in immunity levels caused by changes in melatonin [12] and vitamin D levels [13–15]; (2) variations in host behavior and the frequency of population contact and travel [16], such as the movements associated with school terms and holidays [17,18]; and (3) the survival and transmission abilities of viruses affected by variations in temperature and absolute humidity, and movements of the upper atmosphere [19].

The influenza virus is mainly spread via the respiratory

†These authors contributed equally to this work.
*Corresponding author (email: tianhuaiyu@gmail.com)

tract [20]. Virus present in respiratory secretions is transmitted through spitting and latent infections are spread into the air by coughing and sneezing [21]. The transmission of influenza in temperate regions correlates significantly with seasonal variations [22]. In subtropical Southeast Asia the transmission circle occurs twice a year [23,24], and in tropical regions transmission is closely associated with the rainy season [25,26]. Previous studies have found that temperature, relative humidity [27–31] and absolute humidity [32–34] have great influence on the occurrence and spread of the influenza virus. The environmental conditions of temperature and humidity affect the production and infectivity of virus droplet nuclei and the survival ability of virus [33], thus providing a biophysical explanation of the relationship between the variations of season and environment and the duration and characteristics of influenza epidemics [35]. Understanding the dynamics of these relationships is vital for planning and prioritizing measures for the control and prevention of influenza epidemics in different environments [36].

The peak of the 2009 influenza A (H1N1) pandemic is over but there is still a lack of understanding of the transmission mechanisms, the meteorological conditions suitable for the influenza transmission, and the influence of extreme weather and meteorological anomalies on the outbreaks of influenza epidemics. The objective of this study was to construct a theoretical model of influenza outbreaks based on meteorological epidemiology. We collected all the relevant data relating to the incidence of influenza cases during the recent outbreak. We then obtained all the meteorological parameters thought to influence disease outbreak and transmission using geographic information systems and remote sensing. We analyzed the data using statistical tests to explore the meteorological characteristics of influenza outbreaks, and combining this with the knowledge gained from biology experiments and susceptive-infective-removal (SIR) models we designed a meteorological anomaly model of influenza A (H1N1) epidemics, aiming at providing a scientifically based model that would aid the control and prevention of influenza epidemics in the future.

## 1 Materials and methods

### 1.1 Cases and meteorological data

All the data on influenza A (H1N1) cases reported in Changsha from May 22, 2009 to October 11, 2010 (nearly 99.9% cases concentrated from May 2009 to Jan. 2010, 5477/5483× 100%) were obtained from the Changsha Center for Disease Control and Prevention and the Hunan Provincial Center for Disease Control and Prevention (CDC). The data include information about sex, age, residential address, occupation, date of disease onset and diagnostic criteria for each patient.

Meteorological data from 1951 to 2010 was based on daily observation data at Hunan meteorological sites and on interpolated data (Table 1); kriging interpolation was used to calculate the daily meteorological measurements of the study region from May 22, 2009 to October 11, 2010, for temperature, air pressure, absolute humidity and relative humidity relevant to the occurrence of infections in space and time (Figure 1). Based on the daily series of meteorological data from 1951 to 2009, the thresholds of extreme weather conditions were estimated using the 95th and 5th percentiles. The mean values of the daily meteorological series were calculated from 1951 to 2008 and compared with the records from 2009 to obtain the outliers for analyzing the relationship between the outbreak of epidemics and meteorological anomalies. Taking absolute humidity as an example:

$$\Delta \mathrm{AH} = \mathrm{AH}_{2009} - \overline{\mathrm{AH}}, \tag{1}$$

where $\Delta$AH is absolute humidity (AH) anomaly, $\mathrm{AH}_{2009}$ is the daily records of AH in 2009, $\overline{\mathrm{AH}}$ is the mean value of AH in the same periods from 1951 to 2008.

Analysis of the outbreaks and meteorological conditions during the epidemic periods included the following steps: (1) analysis of the transmission of influenza A (H1N1) epidemics when a meteorological anomaly occurs, (2) classification of the daily infections, calculation of the frequency of different meteorological conditions and the corresponding incidence and level of epidemic, and (3) calculation of the frequency of the "combined effects" of multiple meteorological conditions and their relationship to outbreaks.

### 2.2 Experimental studies on transmission of the virus

Experimental studies found that the ability of the influenza virus to survive and spread among mice varies with temperature, relative humidity and season [37] (Harper [38] and Hemmes et al. [39]). In recent years, in experiments using dolphins, Lowen et al. [28–30] found that the transmission ability of the influenza virus in air varies under different conditions of temperature and humidity (H3N2, Influen A/Panama/2007/99; H1N1, Influen A/Netherlands/602/2009). In these studies the transmission ability of influenza virus under different conditions of temperature, absolute humidity and relative humidity was analyzed in 40 sets of experiments, 24 sets using H3N2 and 16 sets using H1N1 (Figure 2). Logistic regression was used to select a suitable model and to extract epidemiological parameters. The absolute humidity conditions in different experiments were calculated using eqs. (2)–(4) below [40]. According to the experimental results, we know that the transmission ability of influenza A (H1N1) virus is different under different environmental conditions.

$$e_s(T) = e_s(T_0) \times \exp\left(\frac{L}{R_v}\left(\frac{1}{T_0} - \frac{1}{T}\right)\right), \tag{2}$$

**Table 1** Data source and description

| Variables | Data source | Description |
|---|---|---|
| Cases | Changsha Center for Disease Control and Prevention | Case report |
| Temperature/day | China's Meteorological Science Data Shared Services Nets (http://cdc.cma.gov.cn/) | Site data |
| Relative humidity/day | China's Meteorological Science Data Shared Services Nets (http://cdc.cma.gov.cn/) | Site data |
| Air pressure/day | China's Meteorological Science Data Shared Services Nets (http://cdc.cma.gov.cn/) | Site data |
| Absolute humidity/day | Calculated by eqs. (2)–(4) | Site data |
| Temperature distribution/month | http://www.worldclim.org | Interpolation data |
| Precipitation distribution/month | http://www.worldclim.org | Interpolation data |

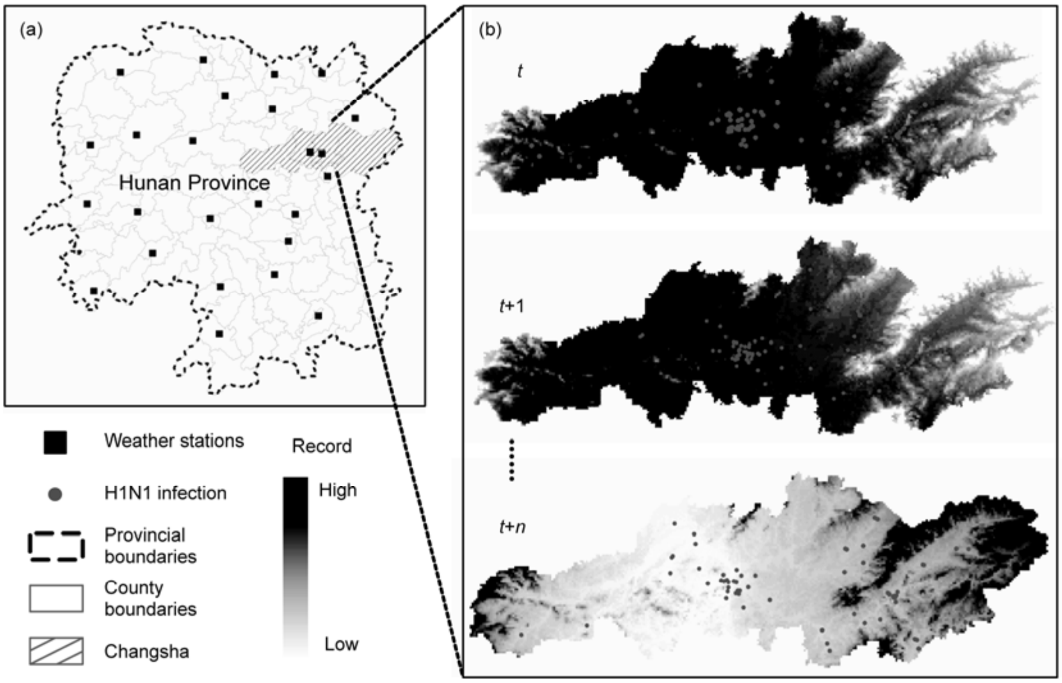

**Figure 1** Daily interpolation of meteorological data and the spatial-temporal records of infections. (a) Meteorological stations in the study area; (b) interpolation of daily meteorological data and the distribution of infections.

$$\varphi = \frac{e}{E} \times 100\% , \quad (3)$$

$$\rho_w = \frac{e}{R_v T} , \quad (4)$$

where $e_s(T)$ is the saturation vapor pressure at the temperature $T$, $T_0$=273.15 K, $e_s(T_0)$=611.29 Pa, $L$ is the latent heat of water evaporation, $R_v$ is the gas constant of water evaporation, 461.5 J kg$^{-1}$ K$^{-1}$; $\varphi$ is relative humidity, $e$ is vapor pressure; $E$ is saturation vapor pressure; $\rho_w$ is absolute humidity.

### 1.3 Meteorological anomaly model in the influenza epidemic outbreak

In this study, the predicted minimum basic reproductive rate ($R_{0\min}$) is determined by the mutation of the virus and the immune levels of the population in the outbreak of influenza epidemic, while the wave of the basic reproductive rate ($R_{0\max}$–$R_{0\min}$) is affected by the meteorological anomaly. Based on experimental research of the transmission ability of influenza virus and the meteorological statistics, analysis of the influence of meteorological anomaly on the spread of influenza was introduced to a susceptive-infective-removal (SIR) model. According to the general infection mechanism, SIR classifies the target population of the disease into three specific groups, namely the susceptible (S), infective (I) and removed (R) individuals. The model has achieved good results and been widely used to predict the dynamics of infectious diseases [41,42].

The basic reproductive rate $R_0$ is equal to the contact rate divided by the removal rate, namely the number of susceptible infected by a single infective case; only when $R_0$>1 can the disease spread among population: if $R_0$<1, the disease will disappear; if $R_0$=1, the disease will remain unchanged, and neither spread nor disappear [43,44]. The meteorological anomaly model can therefore be expressed as

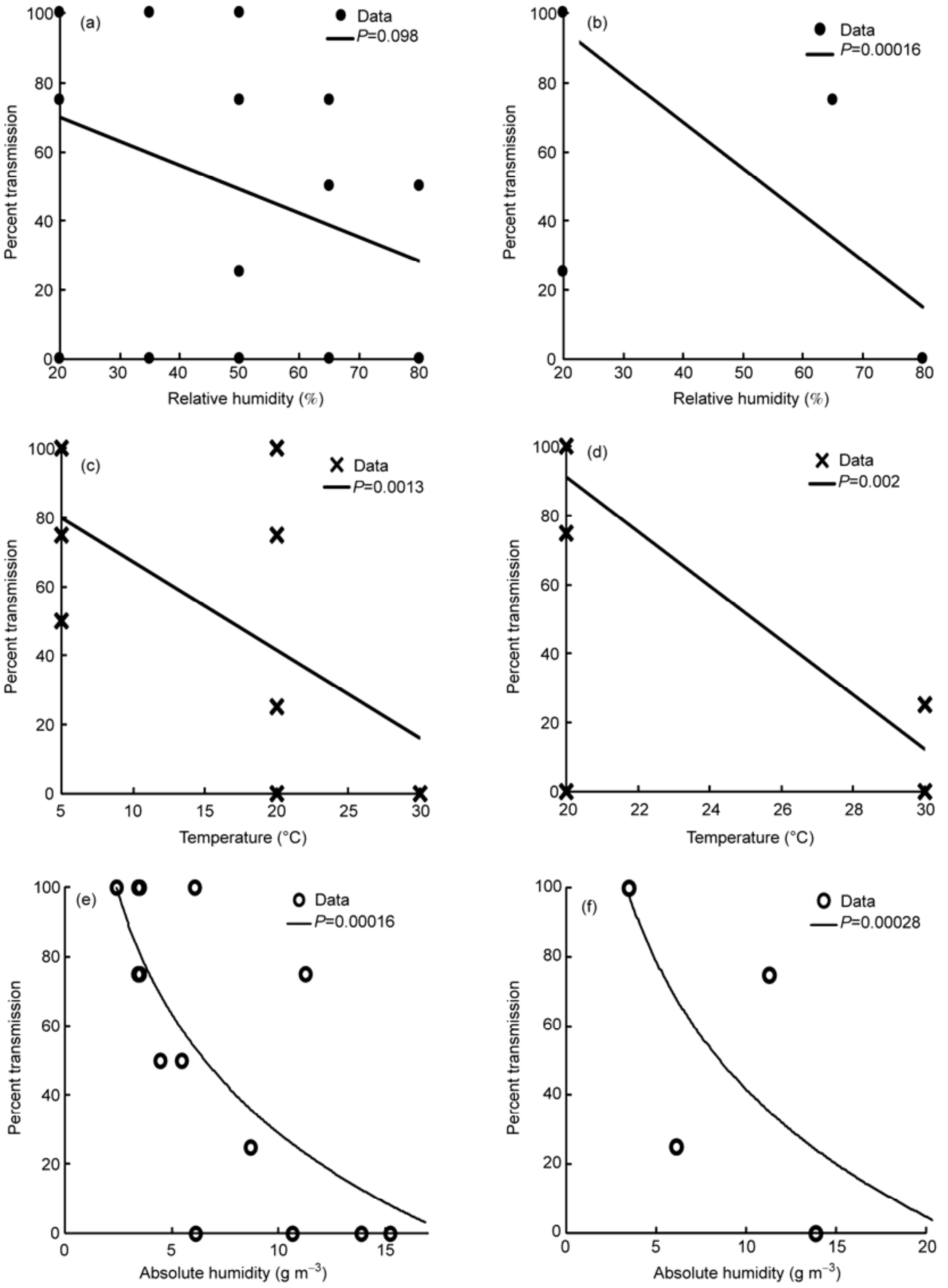

**Figure 2** The experimental results of the transmission ability of influenza virus. Significance of each model fit was assessed by using the *t* statistic for which the *P* value is shown in the legend. (a) Relative humidity regression (H3N2); (b) relative humidity regression (H1N1); (c) temperature regression (H3N2); (d) temperature regression (H1N1); (e) absolute humidity regression (H3N2); (f) absolute humidity regression (H1N1).

$$\frac{\mathrm{d}S(t)}{\mathrm{d}t} = -\frac{\beta(t)I(t)S(t)}{N}, \quad (5)$$

$$\frac{\mathrm{d}I(t)}{\mathrm{d}t} = \frac{\beta(t)I(t)S(t)}{N} - \frac{I(t)}{D}, \quad (6)$$

$$\beta(t) = \frac{R_0(t)}{D}, \quad (7)$$

$$R_0(t) = \exp\left(a \times \left(\Delta \mathrm{AH}(t) + \left|\Delta\rho_{\max}\right|\right) + \log\left(R_{0\max} - R_{0\min}\right)\right) + R_{0\min}, \quad (8)$$

$$\mathrm{AH}(t)=\frac{P(t)\cdot RH(t)\cdot E(t)}{R_v\cdot T(t)\cdot (P(t)-0.378E(t))}, \quad (9)$$

where $S(t)$, $I(t)$, $\beta(t)$ are the susceptible, infective and removed in $t$ day, $N$ is the total population, $D$ is the mean infective stage, $\alpha$ is regression function of experiments of transmission ability of influenza virus (H1N1, Influenza A/ Netherlands/602/2009), $P$ is air pressure, $T$ is temperature, $RH$ is relative humidity, $E$ is saturation vapor pressure at the temperature of $T$, $R_{0\min}$, $R_{0\max}$ is the daily maximum and minimum reproductive, $R_v$ is gas constant of water evaporation, 461.5 J $\mathrm{kg}^{-1}$ $\mathrm{K}^{-1}$, $|\Delta\rho|$ is meteorological anomaly data, if $\Delta\rho$ is max, $R_0=R_{0\max}$, while $\Delta\rho=0$, $R_0$ is the transmission ability under normal conditions.

Simulated annealing (SA) was used to estimate the optimal solution of the model from May 22 to Dec 31 in 2009, the parameters to be estimated including $D$, $R_{0\min}$, $R_{0\max}$ and $\beta$. To predict the epidemic, the cases in 2010 were left as test data, to validate the model effects. The daily new influenza A (H1N1) cases are $obs_i$ ($i$=1,2,3,…, $N$), the prediction of the model is $est_i$ ($i$=1,2,3,…, $N$), objection function "loss" is used to evaluate the predictive effect, and the closer $R^2$ is to 1, the better the predictive results are. In other studies using this type of model, $R_0$ ranges from 1.3 to 3 [10,45–47], and $D$ ranges from 2 to 7 d [45,48]. In this research, $R_{\max}$ ranges from 1.3 to 4, $R_{\min}$ in the model ranges from 0 to 1.3, and $D$ ranges from 2 to 30 d.

### 2 Meteorological analysis and simulation results in influenza outbreaks

#### 2.1 Weather conditions during an influenza A (H1N1) pandemic

The meteorological records for the Changsha City outbreak of Influenza A (H1N1) from October 22 to December 22 show that temperature, absolute humidity, barometric pressure are within the parameters outlined as extreme records (Figure 3). Absolute humidity gave an extremely low value on November 2 (77 cases), November 3 (55 cases), November 17 (184 cases), November 18 (242 cases) and November 19 (245 cases), 2009. With the air being drier the humidity continued to decrease and the incidence of infection gradually increased. Barometric pressure appeared extremely high on November 1–4 (258 cases), November 12–22 (2046 cases), November 28 to December 5 (395 cases), and December 13–21 (51 cases) in 2009. The disease incidence reached its peak in mid-November.

Taking into account that there was considerable variability in weather conditions during the study period, we calculated statistics for the frequency of the various meteorological conditions and compared them with the corresponding case incidence from May 22, 2009 to December 31, 2009. The results showed that over 50% (2929/5439) of the cases appeared in weather when the maximum wind speed was 7–11 km $\mathrm{h}^{-1}$, while 64.9% (3534/5439) of the cases appeared in

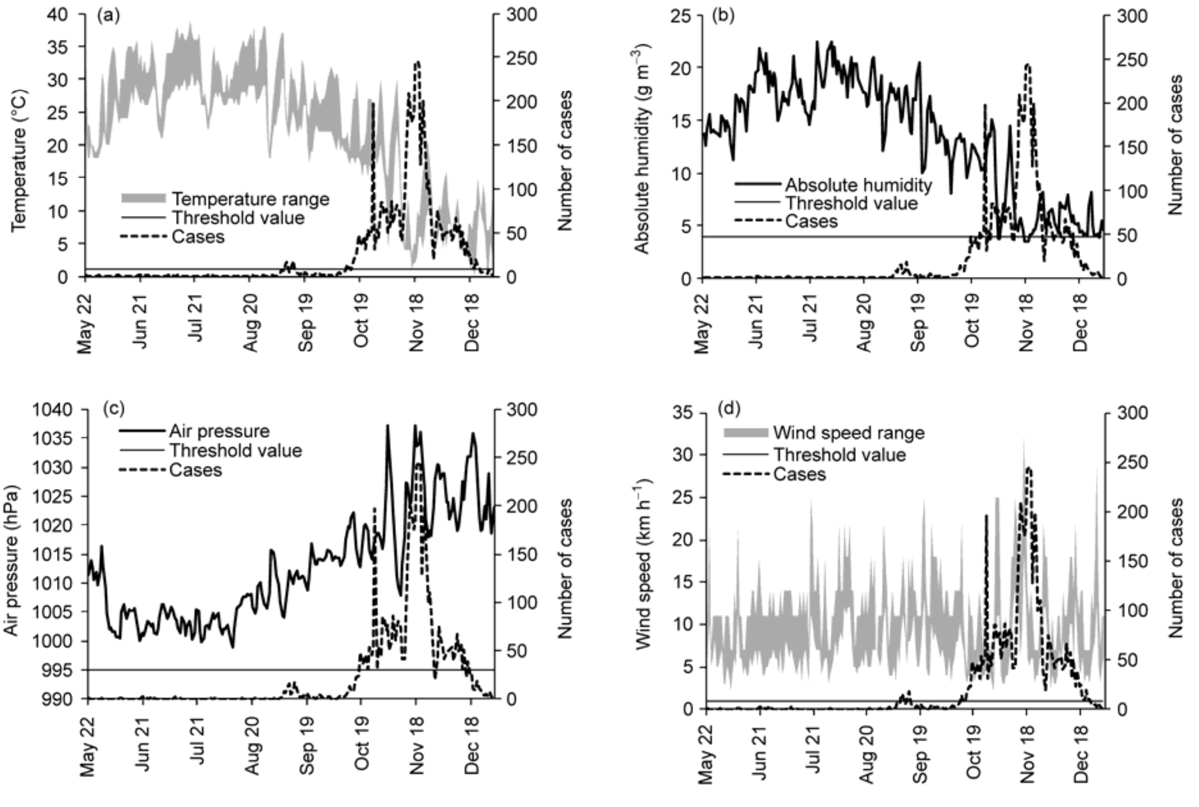

**Figure 3** Weather records and the corresponding prevalence of cases.

weather when the average wind speed was 3–6 km $h^{-1}$, suggesting that a lower wind speed contributed to the spread of influenza A (H1N1). The 3772 cases appeared when the minimum temperature was 0–10°C, accounting for 69.4% of the total number of cases, implying that lower temperatures were more conducive to the spread of the epidemic and 5402 cases (99.3%) were found on days where the pressure was greater than 1008 hPa. As the pressure rises, the probability of cases incidence rises, showing that higher pressure is more conducive to the development of the epidemic. Measurement of absolute humidity appeared to show that the dry air helps the spread of the influenza A (H1N1) since 69.5% cases (3782/5439) occurred when absolute humidity was 3–7 g $m^{-3}$.

Multivariate statistical analysis of the meteorological elements (Table 2) shows that weather conditions of the influenza A (H1N1) epidemic were closely connected with absolute humidity, and barometric pressure. The largest number of cases appeared when weather was composed of the following four meteorological conditions: (1) low temperature, dry, high pressure; (2) dry, low wind speed, high pressure; (3) low wind speed, high pressure; and (4) low temperature, dry, low wind speed, high air pressure. The influenza outbreaks occurred when the temperature was below 10°C, absolute humidity was lower than 10 g $m^{-3}$, wind speed lower than 10 km $h^{-1}$ and air pressure higher than 1010 hPa. Under these circumstances, the high pressure may have allowed virus droplets to sink in the air, the low temperature may have contributed to the survival of the virus, and the low wind speed and dry air may have helped aggregation and thus the spread of the influenza virus. In addition, in the second half of 2009 the influence of El Niño was evident in abnormal weather conditions including temperature, pressure and absolute humidity. Minimum temperatures fell below about 7°C, the absolute humidity was lower than the mean of 5 g $m^{-3}$, and the pressure was higher than the mean of about 20 hPa in Changsha over this period and was significantly correlated with incidence of influenza A (H1N1) (Figure 4).

### 2.2 Simulation results

Iteration of the simulated annealing algorithm obtained an optimal solution of epidemiological parameters: $R_{0\min}$=1.28, $R_{0\max}$=3.20, removal rate=0.17, and average infection period ($D$)=5.88 days (including the invisible infection period). However, the weather anomaly model simulation of the development of H1N1 influenza in Changsha gives a beffer degree of fit (Figure 5). The ($R^2$) of predicted results and actual observations is 0.863. Using the 2010 data this model predicted that the epidemic of influenza A (H1N1) in Changsha would finish at the end of January 2010, which concurred closely with the actual situation and illustrated the strength of the model.

The model's forecasting power appeared erroneous from October 23 to November 1, 2009. Further investigation showed that this was the result of outbreaks in the two schools in the study area. In this case the model failed because, on the one hand, the estimation of basic reproductive number is mostly based on the height of the statistical population, while in isolated small communities $R_0$ is often

**Table 2** Statistical representation of the correlation between the number of cases and days with variable weather conditions[a)]

| Minimum temperature <10°C | Humidity <10 g $m^{-3}$ | Average wind speed <10 km $h^{-1}$ | Barometric pressure >1010 hPa | Outbreak influence cases/day | | | | | |
|---|---|---|---|---|---|---|---|---|---|
| | | | | 0 | 1–9 | 10–49 | 50–99 | 100–99 | >200 |
| | | | | 2 | 1 | 1 | 0 | 0 | 0 |
| o | | | | 0 | 0 | 0 | 0 | 0 | 0 |
| o | o | | | 0 | 0 | 0 | 0 | 0 | 0 |
| o | | o | | 0 | 0 | 0 | 0 | 0 | 0 |
| o | | | o | 0 | 0 | 0 | 0 | 0 | 0 |
| o | o | o | | 0 | 0 | 0 | 0 | 0 | 0 |
| o | o | | o | 0 | 2 | 5 | 3 | 0 | 0 |
| o | | o | o | 0 | 0 | 0 | 0 | 0 | 0 |
| | o | | | 0 | 0 | 0 | 0 | 0 | 0 |
| | o | o | | 0 | 0 | 0 | 0 | 0 | 0 |
| | o | | o | 0 | 0 | 1 | 0 | 0 | 0 |
| | o | o | o | 0 | 2 | 0 | 4 | 1 | 0 |
| | | o | | 75 | 21 | 0 | 2 | 0 | 0 |
| | | o | o | 14 | 24 | 11 | 5 | 0 | 0 |
| | | | o | 0 | 3 | 2 | 0 | 0 | 0 |
| o | o | o | o | 0 | 6 | 13 | 11 | 11 | 4 |

a) o: Under a corresponding climate condition.

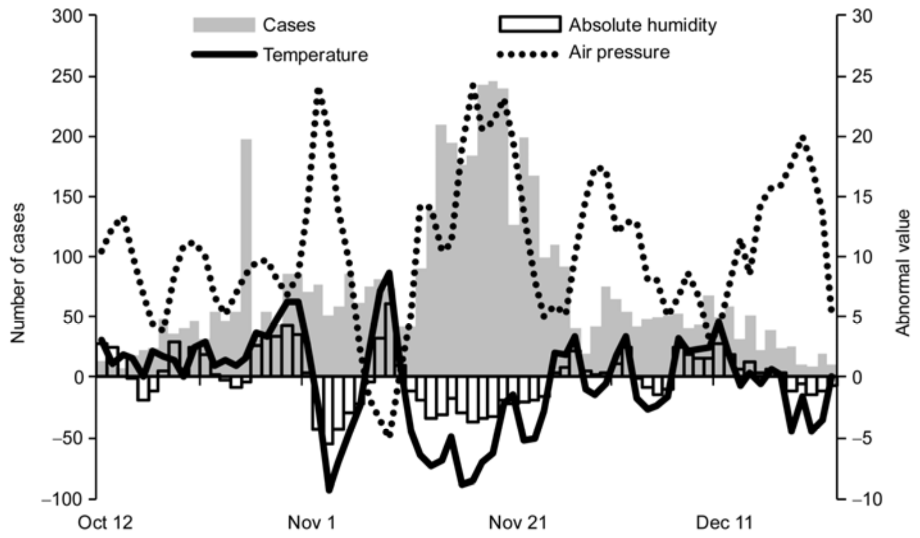

**Figure 4** Abnormal weather and corresponding number of cases during influenza A (H1N1) flu outbreak in Changsha City.

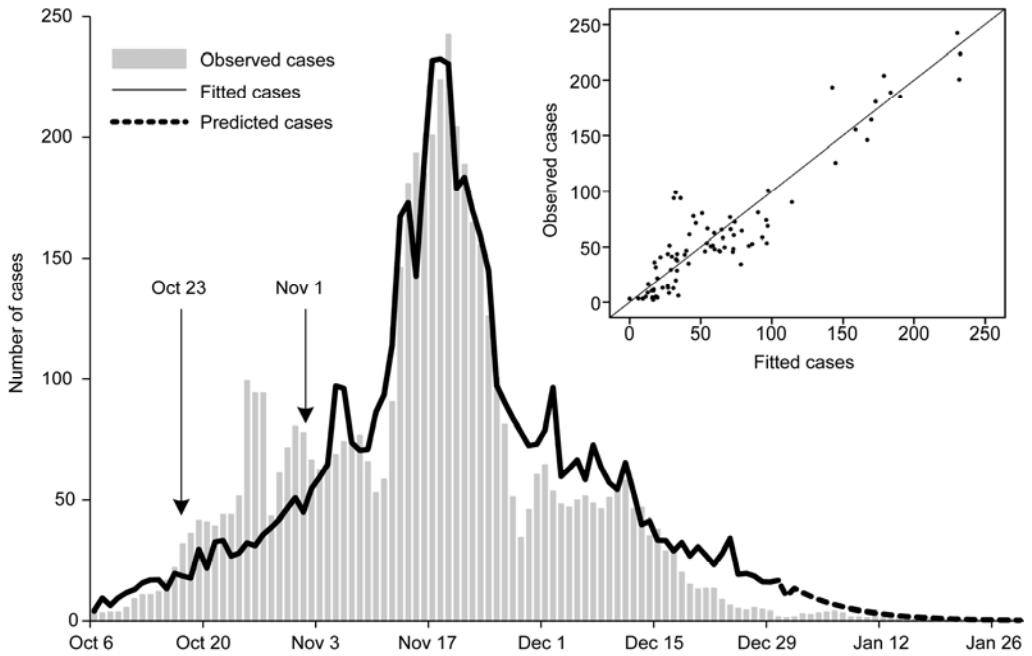

**Figure 5** Daily incidence of influenza A (H1N1) cases in relation to the period of abnormal meteorological conditions in Changsha City, 2009.

larger [49,50], and on the other hand, because in areas with special characteristics such as a school or among troops there is often an abnormally high frequency of susceptible individuals living in close contact.

### 3 Discussion

The occurrence, development and transmission of infectious diseases are affected by pathogen and host, and pathogen and external environment (natural factors and social factors) [20]. It is impossible to understand the whole dynamics of transmission simply by identifying the component parts. By taking a multidisciplinary and integration approach, we can study the occurrence and transmission of infectious disease from a new and much wider perspective. In this study, we found a close connection between weather conditions and H1N1 influenza epidemic pattern in Changsha. On the one hand, extreme or anomalous measurements of single meteorological factors affected the transmission of influenza; on the other hand, large number of influenza cases occurred during periods of extreme or anomalous instances of various meteorological factors. Using experimental studies, meteorological observation and epidemiological models, we can analyze the patterns and explore the dynamics of the laws governing transmission. Through a combination of infectious disease surveillance systems and weather forecasting, valuable early warning systems can be put in place and prevention and control of major influenza outbreaks can be carried out. In addition, especially vulnerable groups, for example, in schools, offices and other crowded indoor environment can be targeted with appropriate methods for reducing the transmission and survival ability of the influenza virus and thus reducing the burden of disease [51]. Different indoor environments will require differing approaches to control and theoretical and practical knowledge should be combined in order to design specific measures that are both economical and feasible for reducing the potential burden of disease.

By statistically analyzing weather conditions associated with influenza A (H1N1), we found that the daily minimum temperature, absolute humidity and barometric pressure have a strong influence on the development of the epidemic situation. More patients appear during periods of the low temperature compared with high temperature, more cases appear when pressure is higher and a large number of cases appear when absolute humidity is lower (AH<16 g $m^{-3}$). In addition, our "combined effect" [52] statistical analysis showed that when different combinations of factors occurred they could affect the epidemic situation further, creating a dangerously increased risk of disease. These factors included low temperatures, dryness, low wind speed, high-pressure and epidemics in Changsha (greater than 200 cases per day) occurred as a result of the combination of such meteorological factors. It is therefore imperative that the combined effects of meteorological factors are studied for meteorological sensitive infectious diseases.

Year 2009 experienced the fifth highest temperatures since 1980. Extreme global weather and climate conditions occurred from June to December the same year as well as a very strong El Niño effect and, the multivariate ENSO index respectively achieved 0.854, 0.929, 0.738, 0.983, 1.039 and 1.084 [53]. At the same time, the El Niño induced climate change correlated strongly with infection in mainland China with a time lag of 3 to 5 months [54]. According to meteorological records during the period of pandemic influenza A (H1N1), a persistently abnormal climate occurred in late September, early October to mid-December at Changsha (in accordance with the time delay cycle). This is similar to the result of El Niño on the French influenza epidemic cycles, and thus provides a new perspective for a comprehensive understanding of the influenza A (H1N1) pandemic events [55]. Accurate interpretation of the trend signal changes in the weather will provide a valuable early warning signal for the prevention of influenza outbreaks.

This study provided insight into the outbreak of influenza A (H1N1) in Changsha particularly in relation to absolute humidity, the contact rates of infected individuals and population changes, although not all influenza pandemics appeared during extreme weather conditions. Extreme weather and abnormal weather may play a role in fueling the spread of influenza. Population immunity levels and the virus mutations also determine the baseline and trend of the epidemic; therefore, the problem was how to reflect the evolution and variability of the virus in the model. One way of improving the model was to employ Markov Chain Monte Carlo methods involving simulated annealing algorithms with the various components although the performance of the algorithm is related to initial parameter value and parameter sensitivity. An additional important elements to consider was the fact that the incidence of influenza is usually higher in lower age groups and among those working in the service industry. Students and manual laborers also have a higher incidence than professional people, and certain special groups with high mobility are an important factor in the continuing spread and proliferation of the disease. The study also found that the composition of the susceptible population [56] had a significant impact on the fit of the model to the results, posing a problem of how to quantitatively evaluate the effect of population heterogeneity in the flu outbreak and improve the predictive power of the models. Future work will concentrate on further refinement of these models.

*We wish to express our thanks to the anonymous referees for their helpful comments on an earlier draft of this article. This work was supported by Hunan Provincial Natural Science Foundation of China (11JJ3119), the Key Discipline Construction Project in Hunan Province (2008001), and the Scientific Research Fund of Hunan Provincial Education Department (11K037).*

1 Chowell G, Bertozzi S M, Colchero M A, et al. Severe respiratory disease concurrent with the circulation of H1N1 influenza. N Engl J Med, 2009, 361: 674–679
2 Morse S S. Pandemic influenza: Studying the lessons of history. Proc Natl Acad Sci USA, 2007, 104: 7313–7314
3 Webster R G, Bean W J, Gorman O T, et al. Evolution and ecology of influenza A viruses. Microbiol Rev, 1992, 56: 152–179
4 Fraser C, Cummings D A T, Klinkenberg D, et al. Influenza transmission in households during the 1918 pandemic. Am J Epidemiol, 2011, 174: 505–514
5 Taubenberger J, Morens D. 1918 Influenza: The mother of all pandemics. Emerg Infect Dis, 2006, 12: 15–22
6 Murray C J L, Lopez A D, Chin B, et al. Estimation of potential global pandemic influenza mortality on the basis of vital registry data from the 1918–20 pandemic: A quantitative analysis. Lancet, 2007, 368: 2211–2218
7 Barry J M, Viboud C, Simonsen L. Cross-protection between successive waves of the 1918–1919 influenza pandemic: Epidemiological evidence from US Army camps and from Britain. J Infect Dis, 2008, 198: 1427–1434
8 Cox N, Subbarao K. Global epidemiology of influenza: Past and present. Annu Rev Med, 2000, 51: 407–421
9 Palese P. Influenza: Old and new threats. Nat Med, 2004, 10: S82–S87
10 Fraser C, Donnelly C A, Cauchemez S, et al. Pandemic potential of a strain of influenza A (H1N1): Early findings. Science, 2009, 324: 1557–1561
11 Morens D M, Folkers G K, Fauci A S. The challenge of emerging and re-emerging infectious diseases. Nature, 2004, 430: 242–249
12 Dowell S F. Seasonal variation in host susceptibility and cycles of certain infectious diseases. Emerg Infect Dis, 2001, 7: 369–374

13 Cannell J J, Vieth R, Umhau J C, et al. Epidemic influenza and vitamin D. Epidemiol Infect, 2006, 134: 1129–1140
14 Cannell J J, Zasloff M, Garland C F, et al. On the epidemiology of influenza. Virol J, 2008, 5: 29
15 Shaman J, Jeon C Y, Giovannucci E, et al. Shortcomings of vitamin D-based model simulations of seasonal influenza. PLoS One, 2011, 6: e20743
16 Brownstein J S, Wolfe C J, Mandl K D. Empirical evidence for the effect of airline travel on inter-regional influenza spread in the United States. PLoS Med, 2006, 3: e401
17 Cauchemez S, Valleron A J, Boelle P Y, et al. Estimating the impact of school closure on influenza transmission from Sentinel data. Nature, 2008, 452: 750–754
18 Cauchemez S, Ferguson N M, Wachtel C, et al. Closure of schools during an influenza pandemic. Lancet Inf Dis, 2009, 9: 473–481
19 Hammond G W, Raddatz R L, Gelskey D E. Impact of atmospheric dispersion and transport of viral aerosols on the epidemiology of influenza. Rev Infect Dis, 1989, 11: 494–497
20 Li L M. Epidemiology. Beijing: People's Medical Publishing House, 2008
21 Tellier R. Aerosol transmission of influenza A virus: A review of new studies. J R Soc Interface, 2009, 6: S783–S790
22 Simonsen L. The global impact of influenza on morbidity and mortality. Vaccine, 1999, 17: S3–S10
23 Wong C M, Yang L, Chan K P, et al. Influenza-associated hospitalization in a subtropical city. PLoS Med, 2006, 3: e121
24 Russell C A, Jones T C, Barr I G, et al. The global circulation of seasonal influenza A (H3N2) viruses. Science, 2008, 320: 340–346
25 Viboud C, Alonso W J, Simonsen L. Influenza in tropical regions. PLoS Med, 2006, 3: e89
26 Shek L P C, Lee B W. Epidemiology and seasonality of respiratory tract virus infections in the tropics. Paediatr Respir Rev, 2003, 4: 105–111
27 Mubareka S, Lowen A C, Steel J, et al. Transmission of influenza virus via aerosols and fomites in the guinea pig model. J Infect Dis, 2009, 199: 858–865
28 Lowen A C, Steel J, Mubareka S, et al. High temperature (30 degrees C) blocks aerosol but not contact transmission of influenza virus. J Virol, 2008, 82: 5650–5652
29 Lowen A C, Mubareka S, Steel J, et al. Influenza virus transmission is dependent on relative humidity and temperature. PLoS Pathog, 2007, 3: 1470–1476
30 Lowen A C, Mubareka S, Tumpey T M, et al. The guinea pig as a transmission model for human influenza viruses. Proc Natl Acad Sci USA, 2006, 103: 9988–9992
31 Schaffer F L, Soergel M E, Straube D C. Survival of airborne influenza virus: Effects of propagating host, relative humidity, and composition of spray fluids. Arch Virol, 1976, 51: 263–273
32 Shaman J, Goldstein E, Lipsitch M. Absolute humidity and pandemic versus epidemic influenza. Am J Epidemiol, 2011, 173: 127–135
33 Shaman J, Kohn M. Absolute humidity modulates influenza survival, transmission, and seasonality. Proc Natl Acad Sci USA, 2009, 106: 3243–3248
34 Shaman J, Pitzer V E, Viboud C, et al. Absolute humidity and the seasonal onset of influenza in the continental United States. PLoS Biol, 2010, 8: e1000316
35 Minhaz Ud-Dean S. Structural explanation for the effect of humidity on persistence of airborne virus: Seasonality of influenza. J Theor Biol, 2010, 264: 822–829
36 Brian H, Birthe B. Aerosol influenza transmission risk contours: A study of humid tropics versus winter temperate zone. Virol J, 2010, 7: 98
37 Schulman J L, Kilbourne E D. Experimental transmission of influenza virus infection in mice. II. Some facotrs affecting the incidence of transmitted infection. J Exp Med, 1967, 118: 267–275
38 Harper G. Airborne micro-organisms: Survival tests with four viruses. J Hyg, 1961, 59: 479–486
39 Hemmes J H, Winkler K C, Kool S M. Virus survival as a seasonal factor in influenza and poliomyelitis. Nature, 1960, 188: 430–431
40 Xiao H, Tian H Y, Zhao J, et al. Study on meteorological factors during the Changsha pandemic influenza A (H1N1) epidemics. Chin J Epidemiol, 2011, 32: 529–530
41 Shi Y L. Stochastic dynamic model of SARS spreading. Chin Sci Bull, 2003, 48: 1373–1377
42 Wang H Y, Rong F, Ke F J, et al. Control dynamics of severe acute respiratory syndrome transmission. Chin Sci Bull, 2003, 48: 2684–2687
43 Chang C Y, Cao C X, Wang Q, et al. The novel H1N1 influenza A global airline transmission and early warning without travel containments. Chin Sci Bull, 2010, 55: 3030–3036
44 Xiao H, Tian H Y, Zhao J, et al. Influenza A (H1N1) transmission by road traffic between cities and towns. Chin Sci Bull, 2011, 56: 2613–2620
45 Mills C E, Robins J M, Lipsitch M. Transmissibility of 1918 pandemic influenza. Nature, 2004, 432: 904–906
46 Ferguson N M, Cummings D A T, Cauchemez S, et al. Strategies for containing an emerging influenza pandemic in Southeast Asia. Nature, 2005, 437: 209–214
47 Ferguson N M, Cummings D A T, Fraser C, et al. Strategies for mitigating an influenza pandemic. Nature, 2006, 442: 448–452
48 Longini I M, Nizam A, Xu S, et al. Containing pandemic influenza at the source. Science, 2005, 309: 1083–1087
49 Vynnycky E, Trindall A, Mangtani P. Estimates of the reproduction numbers of Spanish influenza using morbidity data. Int J Epidemiol, 2007, 36: 881–889
50 Lessler J, Cummings D A T, Fishman S, et al. Transmissibility of swine flu at Fort Dix, 1976. J R Soc Interface, 2007, 4: 755–762
51 Lipsitch M, Viboud C. Influenza seasonality: Lifting the fog. Proc Natl Acad Sci USA, 2009, 106: 3645–3646
52 Tamerius J D, Wise E K, Uejio C K, et al. Climate and human health: Synthesizing environmental complexity and uncertainty. Stoch Env Res Risk A, 2007, 21: 601–613
53 Chen H B, Fan X H. Some extreme events of weather, climate and related phenomena in 2009. Clim Environ Res, 2010, 15: 322–336
54 Lu A G, Ge J P, Pang D Q, et al. Asynchronous Response of Droughts to ENSO in China. J Glaciol Geocryol, 2006, 28: 535–542
55 Viboud C, Pakdaman K, Boëlle P Y, et al. Association of influenza epidemics with global climate variability. Eur J Epidemiol, 2004, 19: 1055–1059
56 Merler S, Ajelli M. The role of population heterogeneity and human mobility in the spread of pandemic influenza. Proc Biol Sci, 2010, 277: 557–565

**Open Access** This article is distributed under the terms of the Creative Commons Attribution License which permits any use, distribution, and reproduction in any medium, provided the original author(s) and source are credited.
